# Effect of probiotic supplementation along with calorie restriction on metabolic endotoxemia, and inflammation markers in coronary artery disease patients: a double blind placebo controlled randomized clinical trial

**DOI:** 10.1186/s12937-021-00703-7

**Published:** 2021-06-01

**Authors:** Jalal Moludi, Hossein Samadi Kafil, Shaimaa A. Qaisar, Pourya Gholizadeh, Mohammad Alizadeh, Hamed Jafari Vayghyan

**Affiliations:** 1 Research Center for Environmental Determinants of Health (RCEDH), Kermanshah University of Medical Sciences, Kermanshah, Iran; 2 Clinical Research Development Center, Imam Reza Hospital, Kermanshah University of Medical Sciences, Kermanshah, Iran; 3 Department of Nutritional Sciences, School of Nutritional Sciences and Food Technology, Kermanshah University of Medical Sciences, Kermanshah, 5166614711 Iran; 4 Drug Applied Research Center, Tabriz University of Medical Sciences, Tabriz, Iran; 5 Chemistry Department, College of Education, University of Garmian, Sulimmania, Iraq; 6 Nutrition Research Center, Faculty of Nutrition, Tabriz University of Medical Sciences, Tabriz, Iran; 7 Faculty of Health, Arak University of Medical Sciences, Arak, Iran

**Keywords:** Coronary artery disease, Lipopolysaccharide, Gut microbiota, Metabolic endotoxmia, Probiotic

## Abstract

**Purpose:**

Alterations in the gut microbiome (dysbiosis) has been associated with increased microbial translocation, leading to chronic inflammation in coronary artery disease (CAD). It has been proposed that modulation of gut microbiota by probiotic might modify metabolic endotoxemia. Therefore, the purpose of this study was to examine the effects of *Lactobacillus rhamnosus* GG (LGG) on endotoxin level, and biomarkers of inflammation in CAD participants.

**Methods:**

This study was a 12-weeks randomized, double-blind, and intervention on 44 patients with CAD. Patients were randomly allocated to receive either one LGG capsule 1.6 × 10^9^ colony-forming unit (CFU) or the placebo capsules for 12 weeks. In addition, all the participants were also prescribed a calorie-restricted diet. Serum levels of interleukin-1β (IL-1β), Toll-like receptor 4 (TLR4), interleukin-10 (IL-10), and lipopolysaccharide (LPS), were assessed before and after the intervention.

**Results:**

A significant decrease in IL1-Beta concentration (− 1.88 ± 2.25, vs. 0.50 ± 1.58 mmol/L, *P* = 0.027), and LPS levels (− 5.88 ± 2.70 vs. 2.96+ 5.27 mg/L, *P* = 0.016), was observed after the probiotic supplementation compared with the placebo. Participants who had ≥2.5 kg weight loss showed significantly improved cardiovascular-related factors, compared to patients with < 2.5 kg weight reduction, regardless of the supplement they took.

**Conclusion:**

These data provide preliminary evidence that probiotic supplementation has beneficial effects on metabolic endotoxemia, and mega inflammation in participants with CAD.

## Introduction

Cardiovascular disease (CVD) is the leading cause of death worldwide, a significant portion of which can be attributed to ischemic heart disease, often as a result of underlying coronary artery disease (CAD) due to atherosclerosis [1]. Despite the enormous growth in knowledge and advances in identifying traditional risk factors such as hypercholesterolemia, homocystinemia, hypertension, and hyperglycemia, which there are many unanswered questions about other cardiovascular risk factors yet [2, 3]. Currently, the scholars have been paid attention to the role of gut microbiota alteration (dysbiosis) as one of the major etiological factors that are involved in the development of CAD [4].

Numerous data have supported the contribution of dysbiosis in the development of CAD by some mechanisms including increased gut permeability and metabolic endotoxemia [5, 6]. This can may be explained by a microbiome-derived lipopolysaccharide (LPS), a major component of the external membrane in gram-negative bacteria. LPS can pass through the intestinal mucosa to enter the bloodstream, and may represent an important mediator of chronic inflammation [7, 8]. Though endotoxaemia is not necessarily equivalent to increasing LPS, many have defined metabolic endotoxemia as “a situation of chronically elevated plasma LPS” [9]. Chronic inflammation following metabolic endotoxemia might be a possible mechanism for the association between dysbiosis and CAD, which is triggered by dysbiosis [10]. LPS triggers toll-like receptors (TLRs) activation, and induces endothelial damage through increasing the expression of surface adhesion molecules such as cluster of differentiation 14 (CD14) on inflammatory cells, as well as, stimulates the release of proinflammatory cytokines [11]. Endotoxin may also induce plaque formation and progression of atherosclerotic lesions, and release of other molecules from endothelial cells involved in the proinflammatory process [12].

Probiotics are well-defined live microbe, which can administrated in adequate amounts and have positively effects on the health of the host by improving or restoring the gut flora [13]. In addition, probiotics exert anti-obesity, anti-inflammatory and anti-oxidative effects [14, 15]. In additions to probiotics help balance keeping of homeostasis in gut microbiota, they have been also considered as a possible treatment for CAD [16]. A small number of studies have explored the effects of probiotics on systemic levels of endotoxin in some clinical settings. Those that have observed the effect of probiotics administration on endotoxin levels and associated metabolic disorders have revealed conflicting results [17, 18]. Probiotics keep preserve integration of gut barrier function and decrease intestinal permeability, which consequently decline endotoxin levels [19].

Weight loss diets and control of traditional risk factors are the treatment choices for CAD in overweight or obesity participants [20]. Calorie restriction (caloric restriction or energy restriction) is a dietary regimen that reduces food intake without incurring malnutrition [21]. Several guiding principles are recommended following a weight loss program to achieve 5–10% weight reduction [22]. Calorie restriction is known not only to reduce body weight but also to improve various cardiovascular risk factors [23]. Although the few animal studies appear to support the fact that dietary intervention leads to significant improvements in endotoxin levels [24]. In addition, the effect of dietary intervention on endotoxin levels has not been studied in human studies yet.

In spite of the broadly reported immune-regulatory properties of probiotics and its benefits in animal and human models, there have been little or no controlled randomized trials testing the effect of probiotic supplementation along with calorie restriction on metabolic endotoxemia in CAD participants. We hypothesized that probiotics with beneficial effects on gut microbiota may improve endotoxemia in CAD subjects. Therefore, the current study evaluated the effects of probiotic supplementation along with calorie restriction on circulating endotoxin level, and inflammation markers in CAD participants.

## Materials and methods

### Participants

We ran a double-blind randomized, placebo-controlled study to inspect the endotoxemia, and inflammation levels in 44 CAD participants. This trial was done at Shahid Madani hospital, as cardiology and heart surgery hospital of Tabriz University of Medical Sciences, Tabriz, Iran. Our study was in agreement with the Helsinki Declaration of the World Medical Association (2000) and was accepted by our local ethics committee of Tabriz University of Medical sciences (IR.TBZMED.REC.1397.184) and also was listed in the Iranian Registry of Clinical Trials (IRCT) (IRCT20121028011288N15). All patients admitted to this hospital with a diagnosis of CAD were considered for participation in the study and screened by a cardiologist for eligibility. The inclusion criteria were as follows: having CAD; maintaining them during the course of the trial; patients without a special diet (e.g. vegetarian or weight-loss diet); and Body Mass Index (BMI) =25–35 kg/m^2^. Patients were excluded if they refused to participate or if they had a history of gastrointestinal disorders, if they had thyroid, renal, pancreatic, or liver diseases; if they were lactating or pregnant; if they are taking antibiotics, probiotics, prebiotics, and inflammatory drugs for 1 month before the intervention or during the intervention. A third party who was blind to the study gave the randomization sequence extracted from allocation software. The contributors, investigators and the medical providers were blinded after assignment to interventions. Informed consent was obtained from all subjects. The flow chart shows that 1 individual dropped out of the study in the intervention group and 2 in the control group.

### Sample size

To calculate sample size, mean (standard deviation; SD) of LPS was used from a previous clinical trial on the effect of prebiotic on endotoxmia of obese patients [25]. Calculated based on a confidence interval (CI) of 95%, and power of 80% in two-sided tests using Power analysis and sample size software (PASS; NCSS, LLC, US) version 15, the sample size was 20 per group, which was increased to 22, considering a probable 10% dropout rate.

### Study design

A blind trial at two levels (researcher, participants), placebo-controlled, randomized, clinical design was used to examine the effect of probiotic supplementation on endotoxemia in patients with CAD. Each qualified contributor was randomly allocated into probiotic or placebo group, according to 1:1 equal proportion rule. The order of random allocation was made by random sequence software. The random numbers were kept by a free person not complicated in the assessment of the patient, or in the data collection and analysis. In the current study, patients were randomly assigned into two groups to receive whichever probiotic supplements (*n* = 22) or placebo (*n* = 22) for 3 months. Both participants and researchers were unaware of the treatment allocation.

### Intervention

All the participants received a moderate calorie restricted dietary plan during 12 weeks’ intervention period. In this study, the program was designed to enable weight loss of 7–10% of weight, at a rate of 0.5–1 kg/wk. throughout the intervention. Calorie intake was planned based on individual features of the participants and with the aim of daily energy restriction (500 kcal fewer than the total energy requirements [TEE] estimated by Mifflin–St Jeor equation). TEE is comprised of Resting Energy Expenditure (REE), Activity Energy Expenditure (AEE) and Thermic Effect of Food (TEF). REE represents the largest proportion of TEE (60 to 75%). AEE accounts for 15 to 30% and TEF for the remaining 10% of TEE. The diets were provided 55 ~ 60% of TEE from carbohydrate, 10 ~ 15% from protein, and 25 ~ 35% from fat. Meal plans were prepared based on these calculations, and according to the food-based dietary guidelines for Iranians (available at http://www.fao.org/nutrition/education/food-baseddietary-guidelines/regions/countries/iran/fr/).

Patients in the probiotic group received one probiotic capsule daily containing a *Lactobacillus rhamnosus (*LGG*))* 1.6 × 10^9^ colony-forming unit (CFU) with their lunch. In the placebo (control) group, the capsules contained maltodextrin (Tak Gen Zist Pharmaceutical Company, Tehran, Iran). The physical properties of the placebo were identical in terms of shape, color, size, packaging, and smell but contained no bacteria. Phone contacts were made to ensure adherence twice a month. Compliance to supplementation was established by requesting participants to return the medication containers. The remaining capsules were counted and subtracted from the number provided to determine the number taken. To increase the compliance, all subjects received short messages on their cell phones reminding them to take the supplements every week. For ethical issues, the subjects were allowable to take their routine medications. However, taking any antioxidants and/or vitamin supplements were prohibited during the trial. The participants were allowed to discontinue the trial if they were unwilling to complete or experience any adverse effect during the intervention. Adverse effect evaluation was complete during the trial by questioning the participants and assessments for any adverse effect related to the intervention. At the beginning of the study, participants were recommended not to change their level of physical activity.

### Dietary assessment

Dietary intake was assessed using a dietary record at month 0, and 3 of the intervention. We used Nutritionist IV software adjusted for Iranian diets to acquire nutrient intakes of participants based on the average of three-day food diaries.

### Physical activity assessment

The physical activity assessment was gotten to monitor patient’s usual physical activity levels throughout the study. The validated short-form International Physical Activity Questionnaire (IPAQ) was used to measure the participant’s physical activity. Based on previous studies, physical activities were classified as low, moderate, and high.

### Assessment of anthropometric indices

Body weight was assessed via a scale with 250 g accuracy (Seca, Hamburg, Germany) and patients were measured while wearing a minimum dress and without shoes. Height without shoes was measured by a tape with 0.5-cm accuracy. BMI was computed by dividing weight (Kg) by height^2^ (m^2^). To avoid measurement bias, all measurements were taken by a trained dietitian.

### Biochemical variables

After an overnight fasting (12 h), blood samples were taken by venipuncture of the antecubital vein, using vacuum tubes. Blood serum was obtained from whole blood through centrifugation at 2500 rpm for 10 min. Fasting blood sugar (FBS) and lipid profile (including High density lipoprotein cholesterol (HDL-C), Low density lipoprotein cholesterol (LDL-C), Triglycerides (TG), and Total cholesterol (TC)) were examined using commercial kits (Pars Azmoon Inc., Tehran, Iran) and a Selectra Pro M autoanalyzer (Vital Scientific, Spankeren, the Netherlands). Endotoxin, and inflammatory markers were measured using enzyme-linked immunosorbent assay [18] kits as follows: Interleukin 1 beta (IL-1β) (intra-assay variation = 5.8%, normal range = 0.02–6 pg/mL, inter-assay variation = 9.06%, sensitivity = 0.01 pg/mL), Toll-Like Receptor 4 (TLR-4) (normal range = 0.05–15 ng/mL, intra-assay variation = 4.58%, inter-assay variation = 7.8%, sensitivity = 0.027 ng/mL), IL-10 (normal range = 0.2–100 ng/dl, intra-assay variation = 10%), and, LPS (inter-assay CV = 10.0%, detectable range = 12.00–1000 ng/ml). Sera Trimethylamine-N-oxide (TMAO), transforming growth factor beta (TGF-β), and matrix metallopeptidase 9 (MMP-9) also were measured via an enzyme-linked immunosorbent assay (ELISA) (Crystal Day, China) as stated by the manufacturer’s information. Serum malondialehyde (MDA) was measured based on reaction with Thiobarbituric Acid (TBA). The number of Bacteroidetes and Firmicutes was measured by Real-time PCR (SYBR green method).

### Statistical analysis

The data were analyzed via SPSS software (version 21; SPSS Inc., Chicago, IL) and the outcomes were stated as mean ± SD. To determine the normal distribution of variables, we used skewness and kurtosis test. Paired samples t-test was applied for within-group comparisons (end-point vs. baseline). Afterward adjusting for the confounders (weight, PAL, and calorie intakes) and baseline levels, we did analysis of covariance (ANCOVA) in which the confounding effect of these variables were taken into account which was used to determine the statistically significant pairwise differences. The analyses were conducted using an intention-to-treat approach [26]. Circulating endotoxin level was intended as a primary outcome, while inflammatory markers were defined as secondary outcomes. For all statistical tests, a *P* value less than 0.05 was interpreted as statistically significant.

## Results

**Fig. 1:**
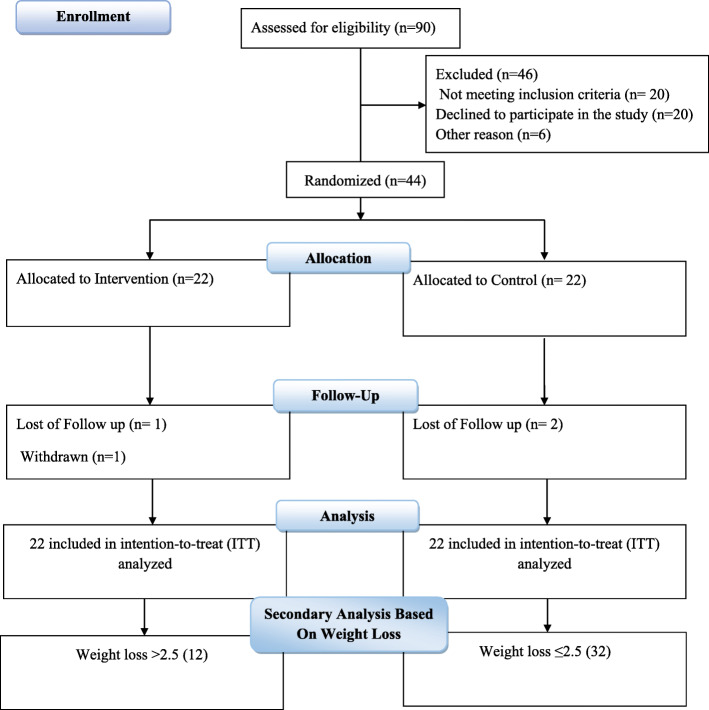
Flowchart of study

Based on the ITT principle, we included all participants (*n* = 44) in analyzes. Figure 1 are shown the study flowchart. Adherence to dosage in our study was over 80% of pills in both groups (Table 1). Overall, 2 participants in of the placebo and 1 in the probiotic group stated symptoms including stomach upset and gastrointestinal problems. These results show that most patients tolerated supplements very well.

**Table 1:** General characteristics of the study subjects

Variable	Probiotic group (*n* = 22)	Placebo group (*n* = 22)	*P*-value
**Age** (years)^a^	56.70 ± 9.10	57.10 ± 7.80	0.876^d^
**Weight** (kg)^a^ at study baseline	75.60 ± 12.30	79.20 ± 12.10	0.390^d^
**Weight** (kg)^a^ after intervention	72.35 ± 12.40	77.54 ± 11.20	0.156
**Smoking**	5 (22)	4 (18)	0.431^c^
**Ejection Fraction** (%)	37.0 (7.1)	38.31 (6.7)	0.527
**Non-Smoking** n(%)	17 (78)	18 (82)	0.431^c^
**PAL** n(%)			0.491^c^
Low	7 (31)	2 (10)	
Moderate	13 (59)	19 (86)	
High	2 (10)	1 (4)	
**Adverse Effects** n(%)	2 (9)	1 (4.5)	0.488
**Compliance Rate (%)**^d^	85	80	0.721

*PAL* physical activity level, *MD* Mean/Median of difference

^a^Values are expressed as mean (SD)

^b^- Values are expressed as frequency (%)

^c^Chi-square test

^d^Independent samples t-test

Baseline medical and demographic data are presented in Table 1. There were no significant differences between the groups regarding weight, BMI, and family history of CAD. At starting point, there was no significant difference between the two groups in term of the level of physical activity. Similarly, physical activity level of the participants who finished the trial stayed unchanged all over the study (*P* > 0.050 for both groups) (data not shown).

Data on dietary intakes (macronutrient distribution, as percentage of calories, changes and dietary fiber) of the patients are summarized in Table 2. Intake of calories and macronutrients were significantly decreased in both groups. We found significant changes in the mean of energy intake of the groups during the intervention (decreasing of − 384.71 and − 284.40 Kcal in probiotics and placebo groups, respectively), however, the between-group differences were non-significant at the end of study (*P* = 0.225).

**Table 2:** Changes in calorie, percent from macronutrients, and dietary fiber throughout the study

Variable	Probiotic group (*n* = 22)	Placebo group (*n* = 22)	Mean difference	*P*-value
**Energy (Kcal/d)**				
Baseline	2214.14 (428.6)	2060.7 (454.38)	153.4 (− 116.1 to 422.8)	0.257^**^
End	1829.34 (174.6)	1776.1 (184.3)	53.27 (− 32.1 to 138.68)	0.225^***^
MD (95% CI), P^*^	−384.71 (− 567.9 to −201.6), **< 0.001**	− 284.40 (− 447.40 to − 121.9), **< 0.001**		
**Carbohydrates (%)**				
Baseline	53.33 (13.43)	54.1 (11.51)	0.80 (− 7.25, 5.78)	0.987^**^
End	54.72 (10.22)	53.88 (11.34)	−0.92 (−7.31, 7.36)	0.807^***^
MD (95% CI), P^*^	1.39 (−7.12, 7.66), 0.667	−0.25 (−6.34, 5.12), 0.672		
**Protein (%)**				
Baseline	13.55 (3.9)	13.99 (3.76)	0.44 (−2.21, 2.21)	0.087^**^
End	15.08 (3.68)	15.7 (2.76)	0.62 (−2.3, 1.14)	0.379^***^
MD (95% CI), P^*^	1.53 (−3.44, 2.21), 0.172	1.71 (−2.21, 0.72), 0.634		
**Fat (%)**				
Baseline	34.12 (9.87)	32.1 (7.72)	−2.01 (−5.03, 8.59)	0.751^**^
End	30.20 (10.62)	31.12 (10.85)	1.01 (−6.23, 5.20)	0.756^***^
MD (95% CI), P^*^	−4.08 (−7.21, 3.33), 0.662	−2.00 (− 4.12, 5.31), 0.656		
**Dietary fiber (g)**				
Baseline	16.40 (4.25)	15.72 (3.17)	−0.68 (−1.2, 3.12)	0.559^**^
End	19.77 (8.05)	17.72 (6.51)	−2.01 (− 2.12, 6.35)	0.342^***^
MD (95% CI), P^*^	3.37 (−0.06, 0.34), 0.090	2.00 (− 0.06, 0.41), 0.147		

Mean (SD) and Mean difference (95% CI) are presented for data

^*^ P based on Paired samples t-test

^**^ P based on Independent samples t-test

^***^ P based on ANCOVA adjusted for baseline values, PAL and weight

Table 3 provides a summary of pre- and post-intervention metabolic endotoxemia and inflammation markers in both groups. Neither the between-group differences nor the within-group variations reached statistical significance for TLR4. A significant decrease in IL1-Beta concentration (− 1.88 ± 2.25, vs. 0.50 ± 1.58 mmol/L, *P* = 0.027), and LPS levels (− 5.88 ± 2.70 vs. 2.96+ 5.27 mg/L, *P* = 0.016) were detected following the probiotic supplementation in comparison to placebo group, respectively. Probiotics administration was resulted in a significant decrease in biomarker levels of inflammation, and metabolic endotoxemia in comparison to placebo group.

**Table 3:** Effect of probiotics supplementation on metabolic endotoxemia and inflammation markers

Variable	Probiotic group (*n* = 22)	Placebo group (*n* = 22)	*P*-value
**IL-10 (**ng/dl**)**			
Baseline	5.11 ± 4.60	4.76 ± 3.60	0.35 (− 2.4 to 2.7), 0.930^**^
End	5.14 ± 2.91	5.78 ± 2.29	0.56 (− 2.6 to 1.6), 0.605^***^
MD (95% CI), P^*^	0.60 (− 1.4, 2.6) 0.542	1.04 (− 1.31, 3.59) 0.399	
**IL1-Beta (**pg/mL**)**			
Baseline	5.62 ± 3.72	5.31 ± 2.09	−0.31 (− 1.4 to 2.6), 0.723^**^
End	3.75 ± 2.10	5.81 ± 3.60	2.05 (− 3.8 to − 0.25), 0.027^***^
MD (95% CI), P^*^	− 1.88 (− 3.25, − 0.48) **0.010**	0.50 (− 1.58, 2.56) 0.546	
**LPS** (ng/ml)			
Baseline	21.92 (11.64)	26.18 (16.85)	4.25 (−13.1, 4.4), 0.335^**^
End	16.04 (6.8)	23.22 (12.22)	7.27 (−13.4, − 1.4), 0.016^***^
MD (95% CI), P^*^	−5.88 (− 10.74, − 1.09), **0.019**	− 2.96 (− 11.6, 5.7), 0.165	
**TLR4** (ng/ml)			
Baseline	11.59 (7.22)	10.58 (8.30)	1.01 (− 2.4, 5.6), 0.765^**^
End	9.8 (7.24)	11.85 (5.6)	2.2 (−5.3, 1.8), 0.301^***^
MD (95% CI), P^*^	−1.79 (−6.21, 3.10), 0.488	1.27 (− 1.7, 4.7), 0.581	

*LPS* Lipopolysaccharide, Mean (SD) and Mean difference (95% CI) are presented for data

^*^ P based on Paired samples t-test

^**^ P based on Independent samples t-test

^***^ P based on ANCOVA adjusted for baseline values, weight, PAL, and calorie intakes

Results stratified by weight reduction of at minimum 2.5 kg was presented in Table 4. Overall, 28% of the participants, who finished the trial had at least 2.5 kg weight loss (8 patients in the probiotic group and 4 patients in the placebo group). Nevertheless, besides of what supplement they receive, participants who reached weight loss had great decrease in some biomarkers in comparison to whom lost < 2.5 kg.

**Table 4:** Changes in biomarkers and variables among the patients who completed the study, stratified by a decrease of 2.5 kg in body weight

Variable	weight loss ≥ 2.5 (*n* = 12)	weight loss ≤ 2.5 (*n* = 32)	MD (95% CI), *P*-value ^a^
FBS (mg/dl)	97.28 ± 20.10	111.52 ± 15.16	−14.33 (− 1.1 to − 27.2), **0**.**032**
Total cholesterol (mg/dl)	140.83 ± 43.16	159.66 ± 47.11	−18.23 (− 13.14 to 50.21), 0.241
TG (mg/dl)	134.34 ± 37.16	160.83 ± 59.25	− 25.52 (− 11.1 to 62.33), 0.174
LDL (mg/dl)	72.36 ± 44.16	87.44 ± 44.39	− 15.11 (− 15.1 to 45.2), 0.326
HDL (mg/dl)	42.00 ± 6.27	44.31 ± 7.59	− 2.31 (− 2.22 to 7.10), 0.353
SBP (mmHg)	113.33 ± 13.18	122.47 ± 12.16	− 9.13 (− 0.26 to 18.31), 0.056
DBP (mmHg)	78.83 ± 7.16	77.10 ± 8.10	− 0.99 (− 8.66 to 6.51), 0.794
BDI	13.50 ± 3.80	16.71 ± 4.33	− 3.21 (1.47to 6.2), **0.035**
EF (٪)	41.75 ± 6.13	39.28 ± 7.23	− 2.46 (− 7.7 to 2.31), 0.301
hs-CRP (mg/dl)	1.14 ± 0.45	1.79 ± 1.25	− 0.65 (0.16 to 0.24), **0.011**
MDA (mg/dl)	125.16 ± 44.16	137.00 ± 72.39	− 11.83 (− 35.1 to 59.71), 0.616
TAC (mg/dl)	219.66 ± 64.83	193.81 ± 89.50	− 25.83 (− 83.1 to 31.32), 0.367
TMAO (mmHg)	23.90 ± 10.18	26.81 ± 33.29	− 2.91 (− 16.1 to 22.2), 0.769
IL1-Beta (mmHg)	4.17 ± 1.93	5.10 ± 3.43	− 0.84 (− 1.21 to 2.94), 0.429
IL-10 (mmHg)	4.05 ± 2.11	6. 09 ± 3.99	− 2.04 (− 0.44 to 4.50), 0.101
MMP (mmHg)	10.47 ± 4.30	16.25 ± 9.75	− 5.74 (− 0.22 to 11.54), 0.059
TGF-Beta	19.50 ± 11.50	20.18 ± 10.42	− 0.77 (− 6.1 to 8.81), 0.833
Procolagen	4.17 ± 1.07	4.50 ± 1.23	− 0.32 (− 1.09 to 0.44), 0.404
LPS (mg/dl)	19.41 ± 11.42	19.68 ± 9.75	− 0.27 (− 6.7 to 7.2), 0.938
TLR4 (mg/dl)	10.11 ± 6.81	11.11 ± 6.39	− 1.00 (− 3.1 to 5.40), 0.650
*L. rhamnosus* (CFU)	181.66 ± 151.83	110.17 ± 123.50	−71.46 (− 161.1 to 18.2), 0.116
Bacteroid (CFU)	0.37 ± 0.27	0.28 ± 0.22	− 0.08 (− 0.24 to 0.07), 0.309
Firmicutes (CFU)	0.23 ± 0.30	0.32 ± 0.31	− 0.08 (− 0.13 to 0.30), 0.777

*FBS* Fasting blood glucose, *BDI* Beck Depression Inventory, *HDL* high density lipoprotein, *LDL* low density lipoprotein, *TG* Triglyceride, *TC* Total cholesterol, *SBP* Systolic blood pressure, *DBP* diastolic blood pressure, *MMP-9* Matrix metallopeptidase 9, *hs-CRP* high sensitivity C-reactive protein, *MDA* malondialdehyde, *TAC* total antioxidant capacity. For other abbreviations see Table 4

Values are expressed as mean (SD)

^a^ Independent samples t-test

## Discussion

As far as we know, this is the first study to provide insight into the anti-inflammatory and anti-endotoxemia effects of probiotic in CAD patients. The results of the current study indicated, for the first time, that weight loss diet along with probiotic supplementation lead to improved endotoxemia, as described by reduction of the LPS levels. In addition, inflammatory markers were influenced favorably by probiotic supplements, among patients with CAD under a calorie-restricted diet. We also found that those who were obedient with the weight loss diet and lost a minimum of 2.5 kg by the end of the intervention had significantly improved metabolic profile in comparison to those with slight compliance with the diet.

In the current study, we ran the weight loss program plus probiotic supplementation. Although weight reduction in the probiotic group was more advantageous than the placebo group, bur this issue did not range to statically significant levels. Similar consequences have been stated with probiotic supplementation in former studies [27]. In the current study, we were statistically observed major changes in nutrient intakes for the period of the intervention and there was a downward trend in the total energy intake in all participants due to the running of weight loss diet for both groups. Though weight loss was similar between the two groups, our results suggest that weight loss diet offers a favorable effect on metabolic disorders, but add-on the diet plus probiotics may promote cardiovascular risk factors (Table 4).

The other result of probiotic supplementation in CAD patients was an improvement in inflammatory markers. To the extent of our knowledge, no study has assessed the effect of probiotics supplementation on endotoxemia in patients with CAD. Former studies have stated microbial dysbiosis in CAD participants [7]. Probiotic supplementation by modulating gut microbiota enhances the immune system function and decreases inflammation and endotoxmia [10, 17]. In our study, we established a statistically significant reduction in LPS levels in the probiotic group in comparison to placebo, but not in TLR4, which suggested probable anti-inflammatory and anti-endotoxemia properties of probiotics. Activation by LPS was resulted in an increase in plasma levels of cytokines like interleukin (IL-) 1 Beta, which was observed in CAD patients [7]. The outcomes of our study show that probiotic supplementation could led to decreased IL-1 Beta and hs-CRP levels. The anti-inflammatory effects of probiotics may involve the production of short chain fatty acids (SCFA) in the gut microbiota and the decreased expression of inflammatory cytokines [28]. This finding is consistent with the outcomes reported by Zarrati et al. [29], who established that *Lactobacillus acidophilus* administration as a probiotic for 2 months resulted in a significant decrease in inflammatory markers among obese participants. In addition, a significant decrease in hs-CRP levels was detected after the intake of probiotic yogurt for 9 weeks among pregnant women [30]. Others have failed to find a significant effect of two-month probiotic supplementation on CRP levels in polycystic ovary syndrome (PCOS) participants [31]. The different results might be clarified by diverse dosages of probiotics supplementations and different clinical setting of individuals, who took part in those studies. Microbial translocation has been proposed to be a driver of inflammation and immune activation in CAD patients [5, 32].

Whereas the contribution of pro-inflammatory cytokines to progression of CAD is well- recognized [33], little is identified about the effect of microbial translocation (increases LPS) on the higher cytokine secretion [34]. To the best of our knowledge, no clinical trial had appraised the effects of probiotics on TLR-4 in meta-inflammation. TLR4 has been suggested to act as a molecular link among LPS, inflammation and the innate immune system [12]. It seems that probiotic supplements reduce inflammation possible via competitive mechanisms, which prevents binding LPS to theTLR4/CD14 complex [10, 35]. In addition, previous studies have indicated that calorie restriction can downregulate LPS-producing enzymes of the commensal bacteria, leading to decreased endotoxemia and inflammation [36]. There is one single study which indicates that being on a very-low calorie diet (800 Cal/day) for 1 month decreases LPS binding protein and zonulin in obese women [37]. Apart from that, there are few clinical trials available about the effects of calorie restriction (25–30% below TEE) on serum LPS. Therefore, we could not compare our results for LPS, to the previous studies about the effect of calorie restrictions on these factors.

As mentioned above, dysbiosis leads to endotoxemia and chronic inflammation. These two factors have been proposed to make participants vulnerable to CADs. Probiotics might reverse these effects by helping to enhance or restore health gut microbiota composition. Cui et al. [38], revealed that probiotic supplementation with *Bifidobacterium spp* in IBD patients for 2 months, meaningfully increased the stool concentration of *Bifidobacterium spp* and *Lactobacillus* spp., compared to placebo. However, in the study of Kato et al. [39], consumption of *Bifidobacterium*-fermented milk for 3 months did not alter the level of stool Bacteriodes.

Our study has some of the limitations, including not assessing the gut microbiota, as well as the duration and doses of the supplementations. Furthermore, the sample size is small, which may interfere with generalizability. So, given the gradually common use of Firmicutes/Bacteriodes ratio, the analysis of these bacterial contents may well be used in the future as indicators of a dysbiotic microbiome that contributes to low grade inflammation and CAD progression. Apart from that, this supplementation is worthy of additional survey.

## Conclusion

The present trial revealed that 12 weeks’ probiotic supplementation (*L. rhamnose*) has a positive effect on endotoxemia and chronic inflammation in CAD patients. In addition, our results propose that calorie restriction plus probiotic supplementations might lead to improved metabolic endotoxemia and better than probiotic supplementation alone. Further studies are needed to replicate the present findings and investigate the mechanisms of the observed effects and test the generalizability of the current findings in CAD patients and patients with other types of cardiovascular disease.

## Data Availability

All data generated and analyzed during this study are included in the manuscript.

## Abbreviations

MI: Myocardial infarction
ELISA: enzyme-linked immunosorbent assay
BMI: Body Mass Index BP Blood Pressure
FBS: Fasting Blood Sugar
HDL: High-Density Lipoprotein
LDL: Low-Density Lipoprotein
TC: Total Cholesterol
TG: Triglyceride
